# Melanin depletion affects *Aspergillus flavus* conidial surface proteins, architecture, and virulence

**DOI:** 10.1007/s00253-024-13107-4

**Published:** 2024-04-09

**Authors:** Ondippili Rudhra, Hariharan Gnanam, Sivaramakrishnan Sivaperumal, VenkateshPrajna Namperumalsamy, Lalitha Prajna, Dharmalingam Kuppamuthu

**Affiliations:** 1 Department of Proteomics, Aravind Medical Research Foundation, Madurai, Tamil Nadu India; 2 Department of Biotechnology and Genetic Engineering, Bharathidasan University, Tiruchirappalli, Tamil Nadu India; 3 Cornea Clinic, Aravind Eye Hospital, Aravind Eye Care System, Madurai, Tamil Nadu India; 4 Department of Ocular Microbiology, Aravind Eye Hospital, Aravind Eye Care System, Madurai, Tamil Nadu India

**Keywords:** Fungal keratitis, Melanin, Melanized conidia, Non-melanized conidia, Surface protein, *G. mellonella*, Kojic acid, *A. flavus*

## Abstract

**Abstract:**

Melanin is an *Aspergillus flavus* cell wall component that provides chemical and physical protection to the organism. However, the molecular and biological mechanisms modulating melanin-mediated host–pathogen interaction in *A. flavus* keratitis are not well understood. This work aimed to compare the morphology, surface proteome profile, and virulence of melanized conidia (MC) and non-melanized conidia (NMC) of *A. flavus*. Kojic acid treatment inhibited melanin synthesis in *A. flavus*, and the conidial surface protein profile was significantly different in kojic acid-treated non-melanized conidia. Several cell wall-associated proteins and proteins responsible for oxidative stress, carbohydrate, and chitin metabolic pathways were found only in the formic acid extracts of NMC. Scanning electron microscopy (SEM) analysis showed the conidial surface morphology difference between the NMC and MC, indicating the role of melanin in the structural integrity of the conidial cell wall. The levels of calcofluor white staining efficiency were different, but there was no microscopic morphology difference in lactophenol cotton blue staining between MC and NMC. Evaluation of the virulence of MC and NMC in the *Galleria mellonella* model showed NMC was less virulent compared to MC. Our findings showed that the integrity of the conidial surface is controlled by the melanin layer. The alteration in the surface protein profile indicated that many surface proteins are masked by the melanin layer, and hence, melanin can modulate the host response by preventing the exposure of fungal proteins to the host immune defense system. The *G. mellonella* virulence assay also confirmed that the NMC were susceptible to host defense as in other *Aspergillus* pathogens.

**Key points:**

• *l-DOPA melanin production was inhibited in A. flavus isolates by kojic acid, and for the first time, scanning electron microscopy (SEM) analysis revealed morphological differences between MC and NMC of A. flavus strains*

• *Proteome profile of non-melanized conidia showed more conidial surface proteins and these proteins were mainly involved in the virulence, oxidative stress, and metabolism pathways*

• *Non-melanized conidia of A. flavus strains were shown to be less virulent than melanised conidia in an in vivo virulence experiment with the G. melonella model*

**Supplementary Information:**

The online version contains supplementary material available at 10.1007/s00253-024-13107-4.

## Introduction

*Aspergillus flavus* and *Fusarium solani* are the predominant pathogens causing fungal keratitis, a corneal infectious disease, affecting many people worldwide (Manikandan et al. 2019; Ung et al. 2019). *A. flavus* keratitis is one of the primary fungal infections affecting mainly the tropical parts of the world. It generates yellowish-green conidia, of much larger size compared to the conidia of *Aspergillus fumigatus*, but still effectively infects human cornea resulting in keratitis (Eisenman and Casadevall 2012; Arunachalam et al. 2022). Melanin is one of the *Aspergillus* species conidia cell wall components present next to the rodlet layer and plays a pivotal role in its pathogenesis (Krishnan et al. 2009; Gow et al. 2017; Smith and Casadevall 2019). Prior studies demonstrated that melanin pigment was covalently bonded to other cell wall components including polysaccharides such as chitin, chitosan, glucans, and plasma membrane-derived lipids (Chatterjee et al. 2015). However, the structure of fungal melanin is less well-defined so far (Nosanchuk et al. 2015) and the microscopical examinations reveal that it has a granular structure overall (Eisenman and Casadevall 2012). Fungal melanin is generally a negatively charged polyphenolic compound, highly heterogeneous, and hydrophobic, and three major types of melanin are produced by fungi (Wheeler and Bell 1988; Pombeiro-Sponchiado et al. 2017). The 3,4-dihydroxyphenylalanine (l-DOPA) and 1,8-dihydroxynaphthalene (DHN) melanins are common in the *Aspergillus* species (Gonçalves et al. 2012; Chamilos and Carvalho 2020). Melanin pigment present on the surface of *A. flavus* conidia significantly enhances the fungal virulence efficiency (Amin et al. 2014). Notably, melanized fungi are more resistant to antifungal agents, host defense, and other environmental stresses compared to non-melanized fungi (Liu et al. 2021). Melanin confers antifungal immunity to *Aspergillus* species by inhibiting Ca^2+^calmodulin mediated LC3-associated phagocytosis (LAP), a non-canonical autophagy pathway (Kyrmizi et al. 2018). *Aspergillus fumigatus* melanin is required for maintaining the integrity of the cell wall and expression of other virulence genes (Pihet et al. 2009). The previous report shows that the l-DOPA pathway was responsible for the melanin pigment production in *A. flavus*, and the l-DOPA melanin pathway inhibitor kojic acid leads to the production of melanin minus conidia (Pal et al. 2014). The tyrosinase enzyme catalyzes the l-DOPA melanin synthesis, and the addition of kojic acid a tyrosinase enzyme inhibitor, in the growth medium leads to the production of pigmentless conidia (Yuan et al. 2020).

The role of melanin on the surface architecture and its function is not well understood in *A. flavus*. In this work, we show that melanin synthesis is inhibited by kojic acid in the clinical isolates of *A. flavus* and the absence of melanin alters surface morphology, hydrophobicity, surface-exposed protein profile, and staining intensity of the conidia. The virulence efficiency of the melanin-negative conidia was assessed using the *G. mellonella* larvae in vivo model (Cutuli et al. 2019).

## Materials and methods

### *Aspergillus flavus* isolates and fungal culture condition

Three *A. flavus* strains, ATCC 200026, MTCC 13369, and MTCC 13368 listed in Table 1, were used in this study. This study was approved by the Institutional ethical committee of Aravind Eye Hospital, Madurai. Clinical strains were isolated from *A. flavus* keratitis patients visiting Aravind Eye Hospital, Madurai, and the clinical samples were collected with the patient’s consent. MTCC 13369 was obtained from a patient whose ulcer healed after antibiotic treatment (healed case) and MTCC 13368 was obtained from a patient who underwent surgery due to non-healing after antibiotic treatment (surgery case). ATCC 200026 (obtained from infected peanut cotyledons) was used as a control. These three strains were identified as *A. flavus*, based on colony morphology, color, and microscopic features on Czapek Dox agar (CZA) and Potato Dextrose Agar (PDA) as described previously (Venkatesh Prajna et al. 2013; Selvam et al. 2015). Subsequently, the identity was confirmed using ITS sequencing. The primers used for ITS region amplification were ITS1 forward: 5′TCCGTAGGTGAACTGCGG 3′ and ITS 4 reverse: 5′ TCCTCCGCTTATTGATATG 3′ (White et al. 1990). *A. flavus* strains were subcultured as described before (Selvam et al. 2015) and the conidia were harvested after seven days of growth at 30 °C on PDA agar plates. A Neubauer counting chamber was used for counting the number of conidia and the conidial suspension was stored in 20% glycerol at − 80 °C.

**Table 1:** *A. flavus* strains used in this study

Strain	Description	Origin and details	Reference
ATCC 200026	Environmental isolate	Peanut cotyledon	www.atcc.org
MTCC 13369	Clinical isolate	Healed case 66 yr. old female; ulcer size: 3.5 mm	http://mtccindia.res.in
MTCC 13368	Clinical isolate	Surgery case 52 yr. old female; ulcer size: 2.6 mm	http://mtccindia.res.in

### Effect of kojic acid on the melanization of *A*. *flavus*

Kojic acid (Sigma-Aldrich, Bangalore, India), an inhibitor of tyrosinase, was used to inhibit the l-DOPA pathway of melanization in *A. flavus*. A mycelial plug of 5-mm diameter was inoculated into a Petri dish containing the PDA culture medium with or without kojic acid (dissolved in 70% dimethyl sulfoxide (DMSO) in different concentrations ranging from 100 to 5 mg/ml. Plates were incubated for 10 days at 30 °C, the growth and pigmentation were recorded as described earlier (Pal et al. 2014). The minimum inhibitory concentration (MIC) values of Kojic acid for three *A. flavus* strains were also determined. After 10 days of incubation, dry conidia were collected and used for further experiments.

### Scanning electron microscopy (SEM)

Dry conidia were transferred to an aluminum specimen mount precoated with sticky carbon conductive adhesive tape and examined with an EVO 18 scanning electron microscope (Zeiss, Oberkochen, Germany). The working temperature of the SEM stage module was − 30 to 50 °C. SEM working conditions were as follows: working distance (WD): 11 mm, electron high tension (EHT): 20.00 kV, signal A: secondary electron (SE1). Several images for each sample were digitally produced and registered at variable magnifications with the Zeiss SEM operating software Smart SEM® V05.06. The statistical analysis was done using the built-in software package in the ImageJ program.

### Lactophenol cotton blue staining

Lactophenol cotton blue stain was prepared by dissolving 20 g phenol crystal, 0.05 g cotton blue, 20 ml lactic acid, 20 ml glycerol, and 20 ml distilled water. A fragment of the fungal colony about two millimeters from the colony edge was removed using an inoculating loop and placed in the drop of stain kept on a clean slide. The slide was closed with a coverslip and incubated for 20 min. After incubation, this preparation was examined under low and high magnification for the presence of characteristic mycelia and fruiting structures using a phase-contrast microscope as described previously (Thomas et al. 1991; Shamly et al. 2014).

### Surface proteins extraction

MC and NMC surface proteins of *A. flavus* were extracted using formic acid as described previously with minor modifications (Paris et al. 2003). Fifty milligrams of both dry melanized conidia (MC) and non-melanized conidia (NMC) were treated for 1 h at 4 °C with 400 μl of 100% formic acid (FA). After incubation, the conidia were centrifuged at 4750 g for 10 min at 4 °C. The supernatant was dried using nitrogen gas and further washed twice with 400 μl of Milli-Q water followed by drying with nitrogen gas. Then, the dried extract was resuspended in 1 × sodium dodecyl sulfate (SDS) sample buffer (2% SDS, 5% β-mercaptoethanol, 10% glycerol in 62 mM Tris–HCl, pH 6.8) and boiled for 15 min. The sample was cooled and centrifuged again at 4 °C 4750 g for 10 min. The clear supernatant containing MC and NMC surface protein extracts was stored at − 40 °C for further use. The protein concentration was determined by the Bradford method (MM 1976) and subjected to sodium dodecyl sulfate polyacrylamide gel electrophoresis (SDS-PAGE) using 16% polyacrylamide gels. After electrophoresis, the proteins were detected by silver staining (Rabilloud et al. 1988).

### Hydrophobicity analysis

The hydrophobicity of MC and NMC was determined using the dry and wet conidia as described earlier (Bom et al. 2015). The conidia were mixed with equal volumes of mineral oil and water, mixed vigorously, and incubated at room temperature for 1 h. After incubation, conidia distribution between water and oil was examined (Bom et al. 2015).

### Calcofluor white staining

Conidia were taken in 0.5-ml Eppendorf tubes mixed with 10 μl of calcofluor white stain, vortexed for 10 s, and then incubated for 15 min in the dark. After incubation, conidia were taken in glass slides, sealed on four sides, and visualized in a fluorescence microscope (40 ×) to check the staining efficiency.

### *Galleria mellonella* infection assay

Sixth instar *G. mellonella* larvae weighing around 160–200 mg were maintained in wood shavings at 20 °C before use. Conidia for the selected isolates (1 × 10^6^ spores/ml) were inoculated in 5 ml of Dulbecco’s Modified Eagle Minimal Medium (DMEM) medium with 5% fetal bovine serum (FBS) and 4 mM l-glutamine and incubated at 37 °C for 6 h. At the end of the incubation period, 10^3^ germinated conidia were suspended in 10 μl and injected into the hemocoel of each larva via the last left pro-leg using a 5-ml insulin syringe. The mortality of the larvae was documented for 3 days. The larval death was evaluated based on the absence of movement in response to stimulation (needle prick), cocoon formation, and melanization of the cuticle as described before (Selvam et al. 2015). Sterile PBS-injected larvae and the untouched larval group were used as controls. Ten to twelve larvae per *A. flavus* strain were used in an experimental set. The survival rate was analyzed using the Kaplan–Meier survival graph.

### Shotgun proteome analysis

The formic acid extracts were separated using 16% SDS-PAGE and stained using Coomassie brilliant blue R-250 (Kawasaki et al. 1990). The samples were electrophoresed until the dye reached one centimeter into the separating gel, and the in-gel-trypsin digestion was done as described earlier (Shait Mohammed et al. 2019a). Subsequently, the proteomic profile was examined using an Orbitrap Velospro mass spectrometer (Thermo, Waltham, MA, USA) as described previously (Shait Mohammed et al. 2019b). In brief, the mass spectrometry data was analyzed using Proteome Discoverer version 1.4 (Thermo, Waltham, MA, USA) using Mascot version 2.4.1.0 (Matrix Science, London, UK) as well as with an inbuilt Sequest HT algorithm. The following parameters were used for database search: peptide tolerance of 10 ppm and fragment tolerance of 0.60 to 0.80 Da. The peptides with a *q*-value lesser than the threshold of 0.01 only were considered for protein identification. Peptides were filtered for high peptide confidence as well as for rank one. Proteins identified by one peptide and at least two peptide spectrum matches (PSM) were taken as reliable identification. Bioinformatics analysis was then performed using the DAVID (https://david.ncifcrf.gov/) gene ontology tool (Huang et al. 2009). The mass spectrometry data were deposited in the ProteomeXchange Consortium (https://proteomecentral.proteomexchange.org/cgi/GetDataset) via the PRIDE partner repository, with the dataset identifier PXD037707 (Perez-Riverol et al. 2022).

## Results

### Inhibition of l-DOPA melanin production in *A*. *flavus* by kojic acid

**Fig. 1:**
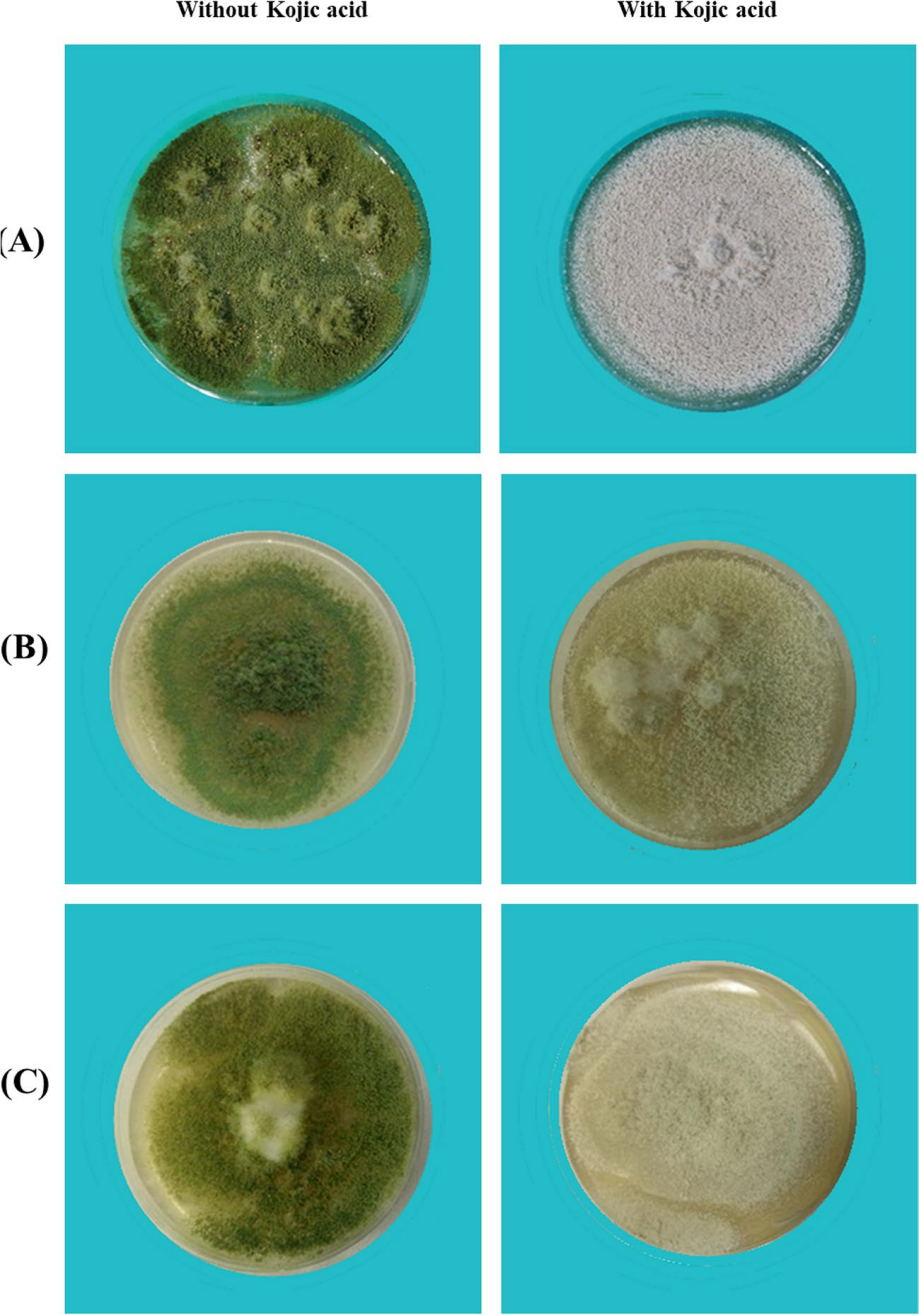
Photographs of *A. flavus* grown in the presence and absence of kojic acid on PDA plates*.* The plates containing kojic acid had 5 mg/ml of the inhibitor. The plates were incubated for seven days as described under materials and methods. **A** ATCC 200026, **B** MTCC 13369, **C** MTCC 13368. In the case of MTCC 13368, the fungal mat was not completely colorless

Kojic acid treatment at the maximum concentration of 5 mg/ml did not affect the growth and sporulation in any of the strains (Fig. 1). However, pigmentation was not seen in any of the isolates grown in kojic acid-containing media. The color of the fungal mat is also distinct in the non-melanized conidia among the three strains. Further, NMC were colorless, smooth, and powdery in texture, whereas MC were dark green, rough, and granular.

### Visualization of MC and NMC surface by scanning electron microscopy

**Fig. 2:**
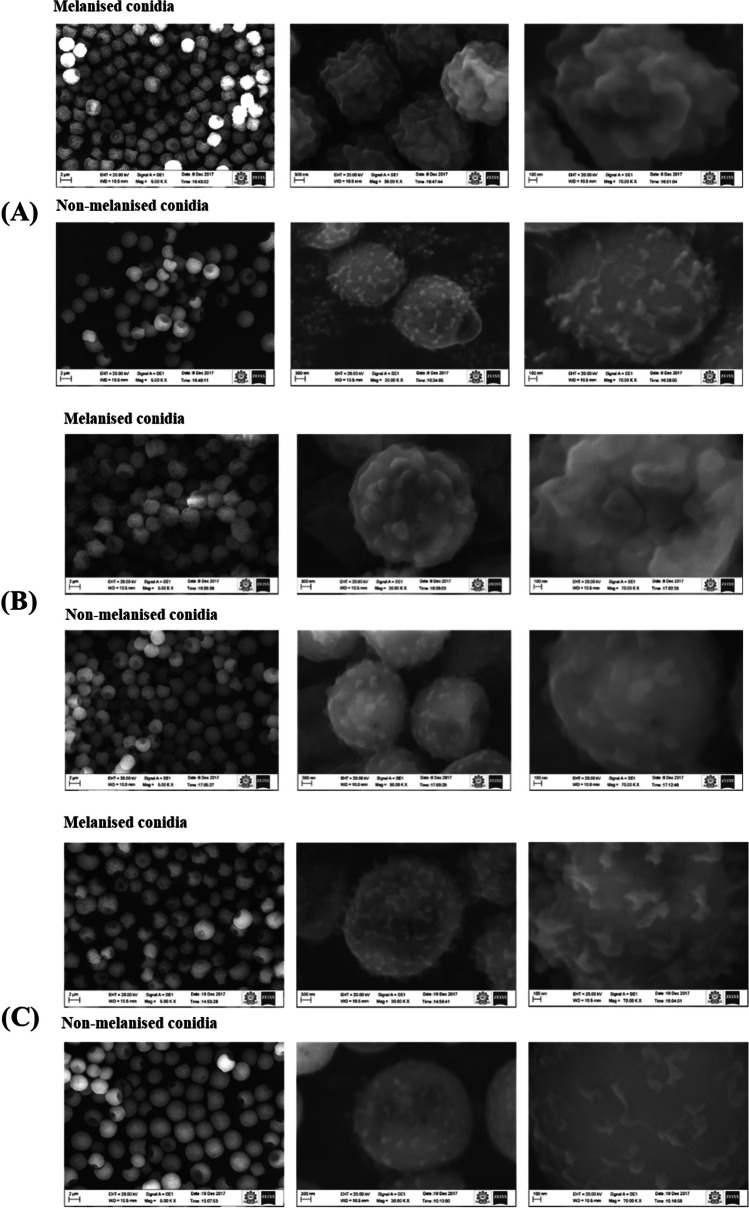
Scanning electron micrographs of melanized and non-melanized conidia. The mean diameter of the conidia measured were melanized conidia 2.83 μm (SD ± 0.114) and non-melanized conidia 3.56 μm (SD ± 0.086). The number of images used for measuring the diameter is shown in Table S1. The altered surface morphology is shown clearly in the higher magnifications. **A** ATCC 200026, **B** MTCC 13369, **C** MTCC 13368. Size bars are shown at the bottom of the figures (length of size bar: **A** 2 μm; **B** 300 nm; **C** 100 nm)

Scanning electron micrographs showed significant changes in the conidial surface between MC and NMC. Data in Fig. 2 show that MC and NMC could be distinguished by their conidial surface morphology. MC presented spiny to finely wrinkled ornamentation, whereas the NMC surface was smooth to distinctly wrinkled. The diameter of multiple conidia was measured using several magnifications of the scanning electron microscope images (Figure S1). The average diameter size (in μm) of the melanized conidia of the three strains are ATCC 200026 (number of conidia measured- 219) 2.82 ± 0.36, MTCC 13369 (number of conidia measured- 201) 3.06 ± 0.32, MTCC 13368 (number of conidia measured- 127) 3.30 ± 0.45, and the diameter of the non-melanized conidia are ATCC 200026 (number of conidia measured- 172) 2.90 ± 0.41; MTCC 13369 (number of conidia measured- 149) 3.14 ± 0.37; and MTCC 13368 (number of conidia measured- 129) 2.94 ± 0.36 (see Table S1). These results show that the size differences are statistically significant. However, further experiments are needed to confirm this data.

The shape of the conidia was another feature that was noted especially at higher magnifications and helped to distinguish between MC and NMC. MC presents predominantly ellipsoidal conidia, while NMC presents globular and sub-globular morphology.

### Evaluation of the lactophenol cotton blue

*Aspergillus flavus* conidia and hyphae appeared pale to dark blue after lactophenol cotton blue staining and different parts of cell-like conidia, hyphae, metulae, phialide, and conidiophores were seen clearly (Figure S2). The conidiophores were variable in length, rough, pitted, and spiny. They covered the entire vesicle, and phialides pointed out in all directions. Conidia were globose to sub-globose, conspicuously echinulate, and even thin delicate hyphae were visualized clearly. There was no significant microscopic morphology difference in these fungal structures between MC and NMC in lactophenol cotton blue staining under these conditions. These results imply that the inhibition of tyrosinase and the absence of melanin did not affect the growth and morphology of *A. flavus* strains.

### Determination of hydrophobicity of non-melanized conidia

**Fig. 3:**
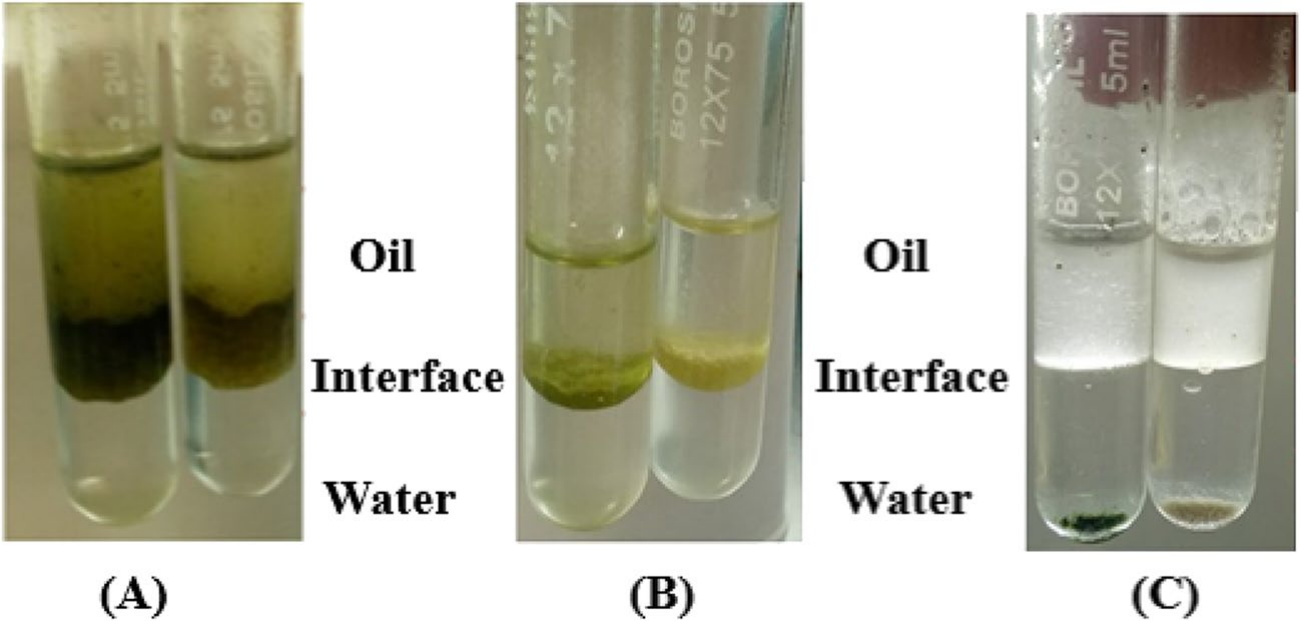
The hydrophobicity of melanized and non-melanized conidia and the effect of hydrophobins on conidial hydrophobicity illustrated by an oil–water two-phase system. Conidia of the clinical strain MTCC 13368 was used in this experiment. **A** Dry melanized and non-melanized conidia bands at the interphase indicate their hydrophobicity. **B** Wet melanized and non-melanized conidia behave similarly to dry conidia and band at the interphase phase. **C** Extraction of hydrophobins using formic acid abolished the hydrophobicity and the conidia settled at the bottom of the water phase

The hydrophobicity of the MC and NM conidia was assessed, and the data are shown in Fig. 3. There was no significant variability in hydrophobicity as the MC and NMC partitioned at the same interphase indicating the presence of an undisturbed outer hydrophobin layer even in the absence of melanin. Formic acid treatment efficiently removed the hydrophobins in both types of conidia and the conidia without the hydrophobins settled at the bottom confirming the above results.

### Investigation of MC and NMC with calcofluor white fluorochrome

**Fig. 4:**
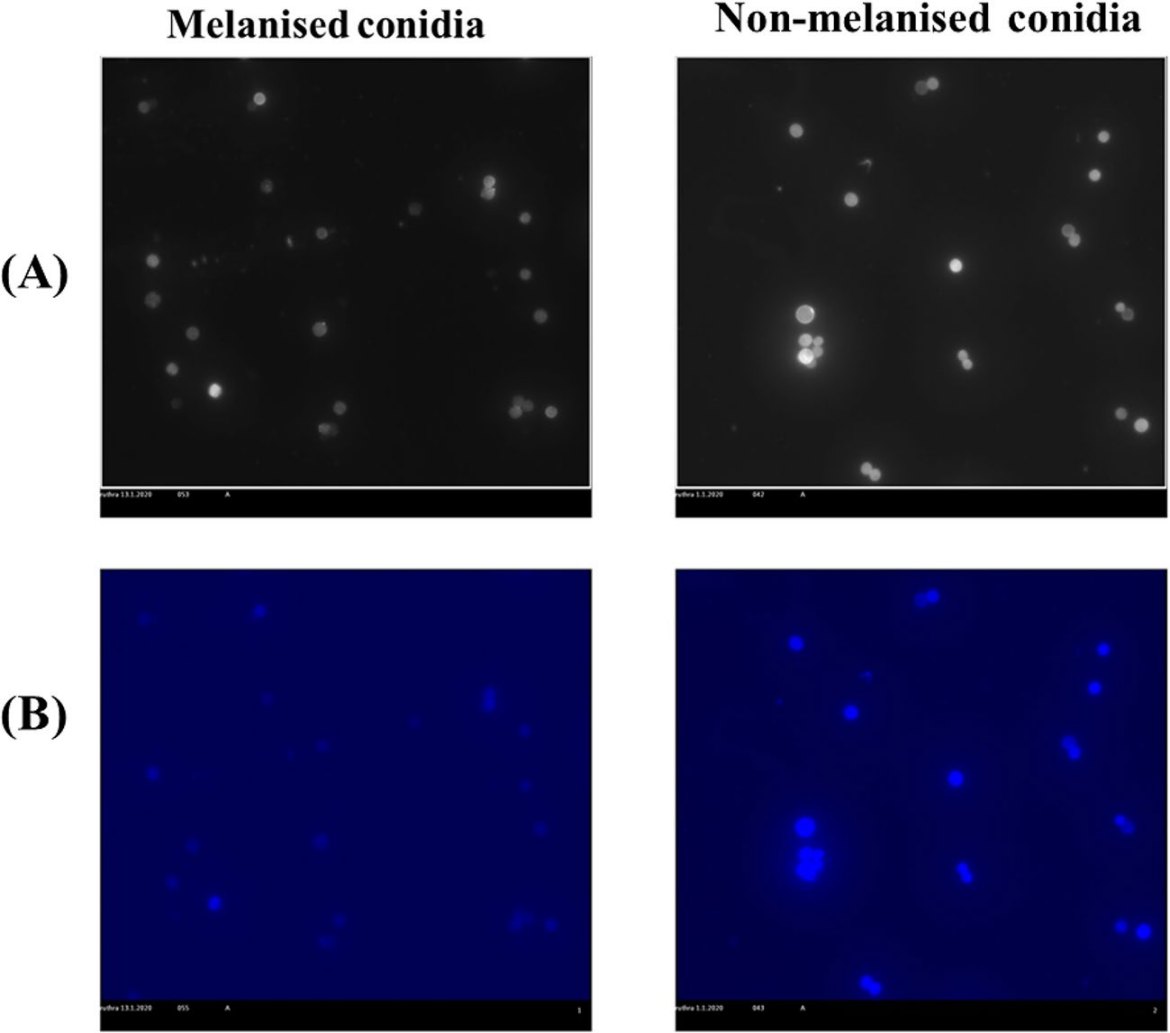
Bright field and fluorescent microphotographs of calcofluor white stained conidia. Conidia of *A. flavus* strain MTCC 13368 were used in this experiment. The top panel is the bright field image, and the bottom panel is the fluorescent image. **A** Melanized conidia, **B** non-melanized conidia. Increased fluorescence of the non-melanized conidia indicated the exposure of the chitin layer in the absence of melanin leading to increased binding of the calcofluor white stain

Calcofluor white stains the fungal conidia by specifically binding to chitin and cellulose of the conidial cell wall (Rasconi et al. 2009)*.* Fluorescence microscopic examination *of A. flavus* MC and NMC stained with calcofluor white (Fig. 4) showed that NMC conidia fluorescence intensity was much higher compared to that of MC. The NMC appeared bright blue, whereas MC were light blue and NMC could be easily differentiated from MC because of the bright blue fluorescence. This indicates that the removal of the melanin layer exposed the chitin layer to the stain.

### In vivoanalysis of MC and NMC virulence efficiency

**Fig. 5:**
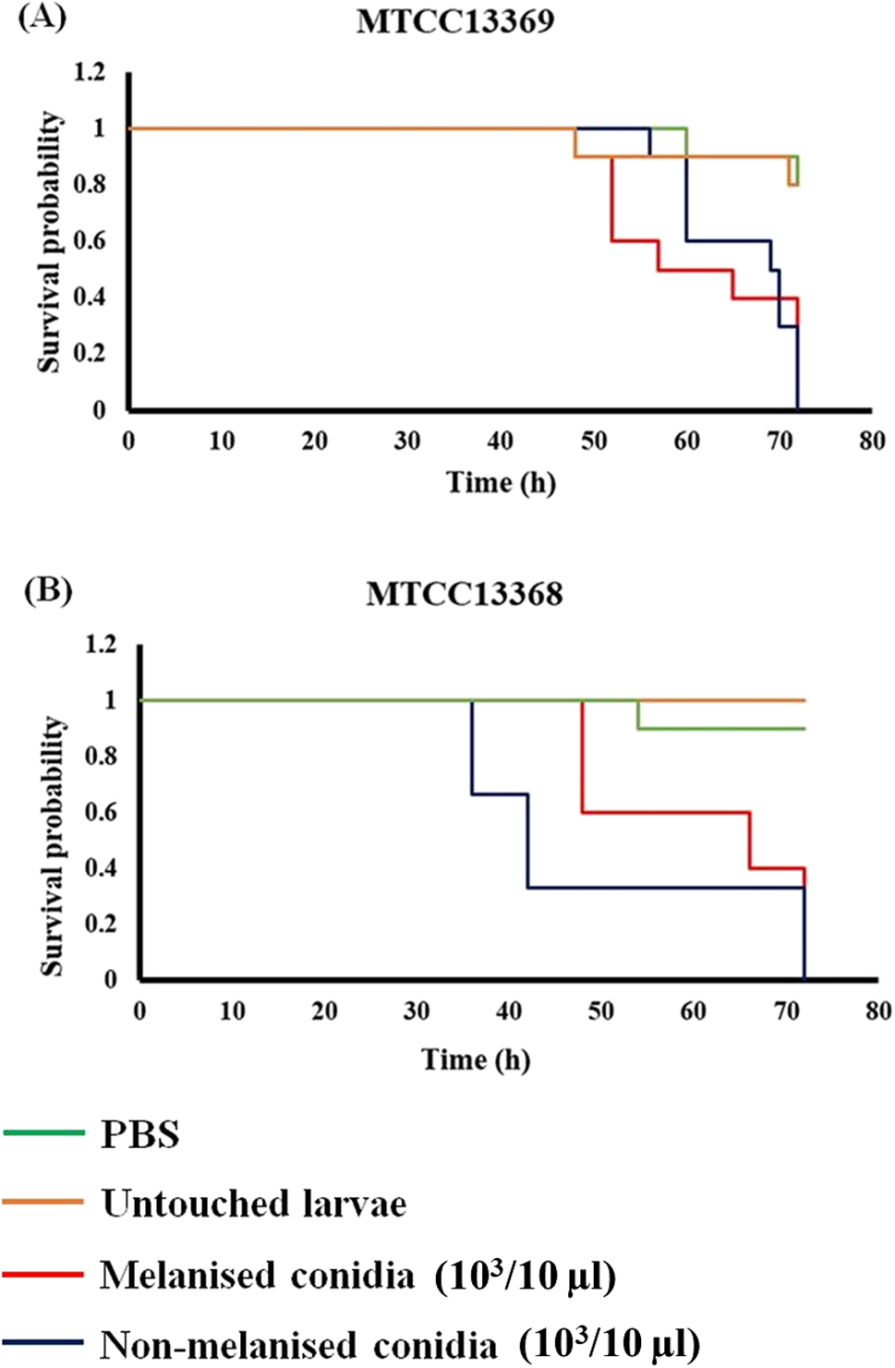
In vivo virulence assay using *G. mellonella* larvae. Kaplan–Meier survival graph showing the effect of melanin on the virulence of the *A. flavus* conidia (10^3^/10 μl). Survival was measured as described under materials and methods. *A. flavus* strains used are **A** MTCC 13369 and **B** MTCC 13368. PBS-injected and untouched larvae used were as controls. Larvae infected with non-melanized conidia survived longer compared to those infected with melanized conidia

The optimal concentration used for the assessment of killing efficiency as described previously (Selvam et al. 2015). In the experiments described, 10^3^ conidia in 10 μl were used as the final inoculum in all experiments (Figure S3). After 72 h of incubation, the survival of the larvae was determined (Fig. 5). We did not find any difference in the survival of *G. mellonella* infected with the conidia with or without melanin from the ATCC 200026 (Figure S4). However, in the case of clinical isolates MTCC 13368 and MTCC 13369, the injected larvae group survived longer compared to the melanized conidia-infected group. In these experiments, each experimental group had 8–10 larvae and the experiment was repeated at least three times. Larvae injected with MC started showing an intense black body color earlier than the NMC-injected larvae, indicating the growth of melanized conidia inside the larvae (Fig. 5). There was a significant visual difference in the color of the larvae injected with melanized conidia, survival duration of larvae, and mortality, between the MC and NMC-injected larvae groups. These results show clearly that the melanized conidia of the clinical isolates were more virulent in this model system. Interestingly, the melanization of the conidia of the saprophyte did not confer any significant advantage in killing the larvae. More studies are needed to understand this.

### Conidial surface protein profile

**Fig.6:**
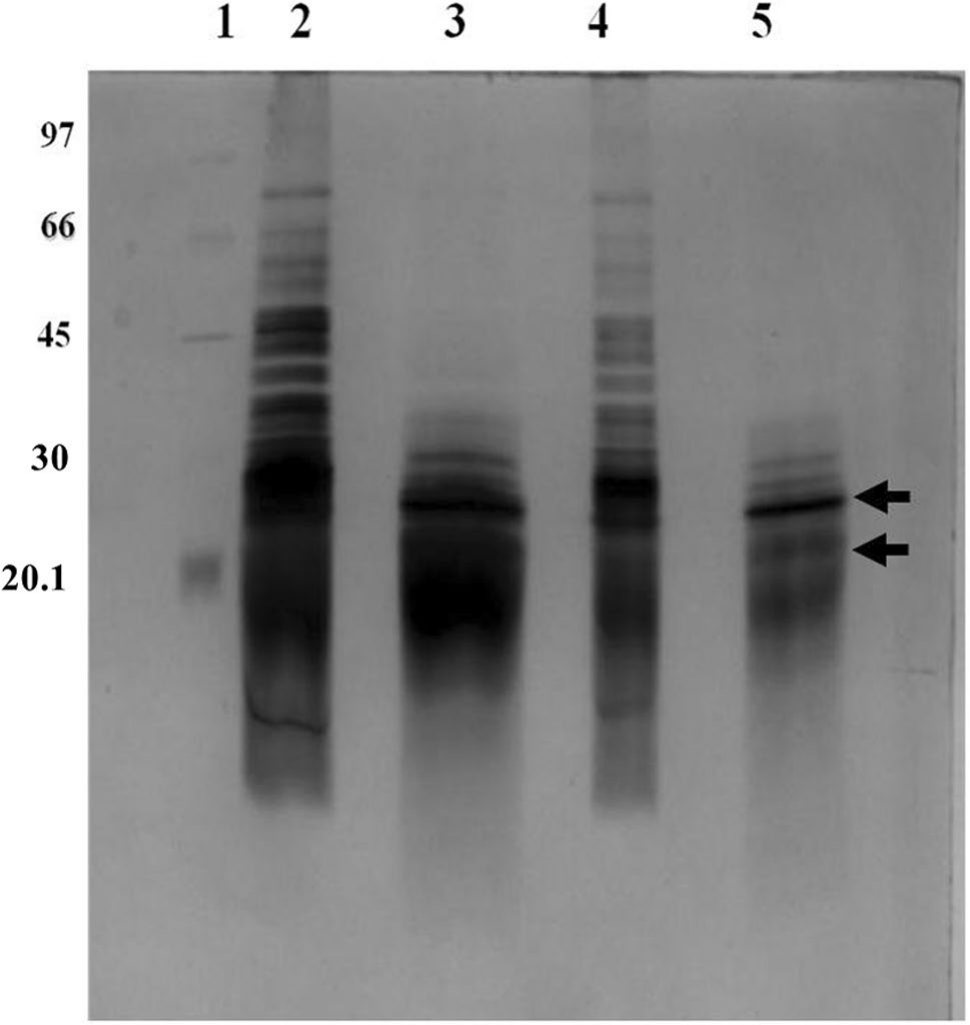
Polyacrylamide gel electrophoresis analysis of formic acid extracts of the conidia. *Aspergillus flavus* strain MTCC 13368 was used in this experiment. The gel was post-processed using silver stain as described under materials and methods. The arrows indicate the hydrophobin proteoforms. Lane 1: Protein molecular weight marker. Lane 2: Non-melanized conidial extract (10 μg). Lane 3: Melanized conidial extract (10 μg). Lane 4: Non-melanized conidial extract (5 μg). Lane 5: Melanized conidial extract (5 μg)

The total cell surface proteins were extracted using 100% FA and separated using SDS-PAGE. Figure 6 shows a sliver-stained gel image of *A. flavus* surface proteins. Two conidial surface proteins with molecular masses of 16 and 14 kDa were found in the MC conidia of MTCC 13368. Previous studies from our group have shown that these two bands represent two proteoforms of hydrophobin A encoded by the *rod A* gene of *A. flavus* (Shait Mohammed et al. 2019a, b). Apart from these two protein bands, more conidial surface protein bands were found in NMC compared to MC. These results indicate that more conidial surface proteins were extractable from conidia without melanin, unlike the ATCC 200026 in which only two hydrophobin bands are found in the FA extracts.

### Comparison of cell wall proteome between MC and NMC

A total of 652 proteins were identified from the extracts of non-melanized conidia, whereas only 260 proteins could be identified from melanized conidial extracts (Table S2). Sixty percent of the proteins identified from melanized conidia were found in the non-melanized conidial preparations also. Hydrophobin, α-1,2-mannosidase family protein, GPI-anchored cell wall organization protein Ecm33 (Yoshimi et al. 2016) and cell wall remodeling enzymes (exo-β-1,3-glucanase exg0, chitinase, 1,3-β-glucanosyltransferase, probable β-glucosidase A, cell wall integrity signaling protein Lsp1/Pil1) (Mouyna et al. 2013; Foderaro et al. 2017) were identified from both NMC and MC. Furthermore, proteins have proven to be involved in the resistance to oxidative stress, including superoxide dismutase (SOD), catalase (Angelova et al. 2005), thioredoxin reductase (Binder et al. 2020), cytochrome c oxidase (Srinivasan and Avadhani 2012), and molecular chaperone Hsp70 (Tiwari et al. 2015). In addition, melanin pigment biosynthesis pathway proteins such aspolyketide synthase, conidial pigment biosynthesis oxidase Arb2/brown2, multicopper oxidase, and laccase (Tsai et al. 1999; Janusz et al. 2020) were also found. Chitinases, 1,3-β-glucanosyltransferase, exo-β-1,3-glucanase Exg0, superoxide dismutase, and thioredoxin reductase are all cell wall proteins that have already been reported to be involved in the virulence of the fungus (Gow et al. 2017).

The total number of proteins identified in NMC was higher than in MC. Tyrosinase enzyme (Yuan et al. 2020), endo-1,3(4)-β-glucanase (Free 2013), alcohol dehydrogenase (Grahl et al. 2011), autophagy-related protein 26 (Zhu et al. 2019), leucine-rich repeat domain protein (Ng and Xavier 2011), and some other proteins were identified only in the NMC surface proteome. Most of the identified proteins in NMC are involved in virulence, stress response, and metabolism (Gow et al. 2017).

## Discussion

The role of the DHN melanin is extensively studied using the pathogenic fungus *A. fumigatus* (Langfelder et al. 2003; Thywißen et al. 2011). C-type melanin sensing receptors (MelLec) found in endothelial cells of mice and the myeloid cells of humans mediate antifungal defense (Stappers et al. 2018; Smith and Casadevall 2019). However, in many cell types, DHN melanin actively inhibits phagosome killing by multiple mechanisms (Schmidt et al. 2020). In this study, we examined the role of l-DOPA melanin of *A. flavus* in antifungal defense and in the organization of the conidial cell wall. l-DOPA pathway-specific inhibitor kojic acid inhibited the production of melanin in the environmental as well as in clinical isolates of *A. flavus* confirming the previous results (Pal et al. 2014). However, there is a conflicting report that a copper-transporting ATPase gene is responsible for *A. flavus* conidial pigment formation (Chang et al. 2019). In our experiments, the inhibitor at the concentration used (5 mg/ml) inhibited melanin formation completely. The discrepancy in the published results could be due to the strains used or the experimental conditions. The polyketide synthase (pks) gene is one of the key genes involved in the *A. fumigatus* DHN melanin pathway and pairwise sequence alignment of the pks gene (CAA76740.1) of *A. fumigatus* and *A. flavus* (QRD90477.1) revealed that 73% DNA sequence identity exists between two species (Chang et al. 2020). Further, the absence of melanin in culture filtrates in all the *A. flavus* isolates (data not shown) indicates that melanin is always cell wall-associated (Eisenman and Casadevall 2012).

Melanin is located beneath the outermost cell wall rodlet layer composed of hydrophobins in *Aspergillus* conidia (Valsecchi et al. 2017). Melanin masks the other immunologically active protein and carbohydrates on the conidial surface and prevents the immune system effectors from recognizing the conidial antigens (Heinekamp et al. 2013). SEM analysis revealed that the MC and NMC surface morphology also changed. Marginal change in the diameter of the spores in NMC spores implied the role of the melanin layer in maintaining the volume and integrity of spores. This is the first SEM report of the MC and NMC morphology of *A. flavus* isolates. Formic acid is shown to extract specifically the hydrophobins of dry conidia of *A. fumigatus* and our data showed the presence of two bands corresponding to the molecular mass of 14 and 16 kDa in the FA extracts of MC (Shait Mohammed et al. 2019b). However, in the NMC extracts, several additional proteins were found. This data shows that several proteins that were buried deep were exposed in the absence of a melanin layer. These two sets of data confirm the disruption of the melanin layer leading to the alteration of the surface architecture and the number of surface-exposed proteins.

Fungal spores maintain a high level of hydrophobicity through their rodlet layer (Wösten and De Vocht 2000). Both MC and NMC spores were hydrophobic in our assay indicating the absence of the melanin layer does not affect the hydrophobin layer in a major way, except for the increase in spore volume. However, the removal of the rodlet layer by FA completely abolished the hydrophobicity of the spores with or without the melanin layer. This data indicates that the integrity of the spore is not completely disrupted in the absence of the melanin layer. *Aspergillus flavus* conidiophores have several distinct structures, and lactophenol cotton blue-stained NMC and MC conidiophores equally well. Conversely, the.

*Aspergillus flavus* MC and NMC examined with calcofluor white stain (Harrington and Hageage 2003; Rasconi et al. 2009) showed that fluorescence was much more intense in NMC than in MC. This shows in the absence of melanin the chitin layer which binds to calcofluor is more accessible to the stain confirming the absence of melanin.

The cell wall-associated surface proteins are essential for fungal pathogenesis (Karkowska-Kuleta and Kozik 2015). Comparative cell surface proteome data showed clearly that many cell surface protein levels were affected in non-melanized conidia. NMC proteome has elevated levels of cell wall remodeling enzymes, immune response proteins, stress-induced enzymes, and enzymes associated with metabolism as well as virulence compared to the MC. This result agrees with our previous *A. flavus* transcriptome data (Arunachalam et al. 2022). The altered proteome is likely due to the alterations in the architecture of NMC cell surface. Functional analysis of these proteins will be important to understand the role of these proteins in host–pathogen interaction.

*Galleria mellonella* infection virulence assay results demonstrated that *A. flavus* MC killed more larvae than its NMC and proved that melanin might be a potential virulence factor for *A. flavus*. It has been shown already that *A. fumigatus* virulence is mainly mediated by DHN melanin, which cause apoptosis inhibition in macrophages and neutrophil by stimulating the phosphoinositide-3 kinase (PI3K)/Akt survival signaling pathway (Volling et al. 2007; Amin et al. 2014; Williams et al. 2021). In contrast, in the case of *Cryptococcus neoformans*, melanin has a decreased virulence in the *G. mellonella* model (Eisenman et al. 2014).

In conclusion, these findings will provide some insights about melanin biosynthesis, its function, and its effects in *A. flavus*, which will help to improve future research on *A. flavus* pathogenicity.

## Supplementary Information

Below is the link to the electronic supplementary material.

Supplementary file1 (XLSX 72 KB)

Supplementary file2 (XLSX 172 KB)

Supplementary file3 (PDF 887 KB)

## Data Availability

This paper and its supplementary information both contain all the data necessary to support the study’s conclusions. The proteomics data were submitted to the ProteomeXchange Consortium (https://proteomecentral.proteomexchange.org/cgi/GetDataset) via the PRIDE partner repository, with the dataset identifier PXD037707.
