# Supplementary material for: Melanin depletion affects Aspergillus flavus conidial surface proteins, architecture, and virulence
Source: Appl Microbiol Biotechnol. 2024 Apr 9;108:291. doi: 10.1007/s00253-024-13107-4 (PMC11004046; doi:10.1007/s00253-024-13107-4)
Supplement: Supplementary file 1 [file 253_2024_13107_MOESM3_ESM.pdf]

Supplemental Material

Journal Name: Applied Microbiology and Biotechnology

Title: Melanin depletion affects *Aspergillus flavus* conidial surface proteins, architecture and virulence.

Names of the Authors: Ondippili Rudhra[1], Hariharan Gnanam[1], Sivaramakrishnan Sivaperumal[2], Venkatesh Prajna Namperumalsamy[3], Lalitha Prajna[4],Dharmalingam Kuppamuthu[1]

Affiliations & Address:

[1]Department of Proteomics, Aravind Medical Research Foundation, Madurai, Tamil Nadu, India.

[2]Department of Biotechnology and Genetic Engineering, Bharathidasan University, Tiruchirappalli, Tamil Nadu, India.

[3]Cornea Clinic, Aravind Eye Hospital, Aravind Eye Care System, Madurai, Tamil Nadu, India.

[4]Department of Ocular Microbiology, Aravind Eye Hospital, Aravind Eye Care System, Madurai, Tamil Nadu, India.

Correspondence: Dr. K. Dharmalingam,

Email Id: dharmalingam.k@gmail.Com

Phone.No: 9443148447

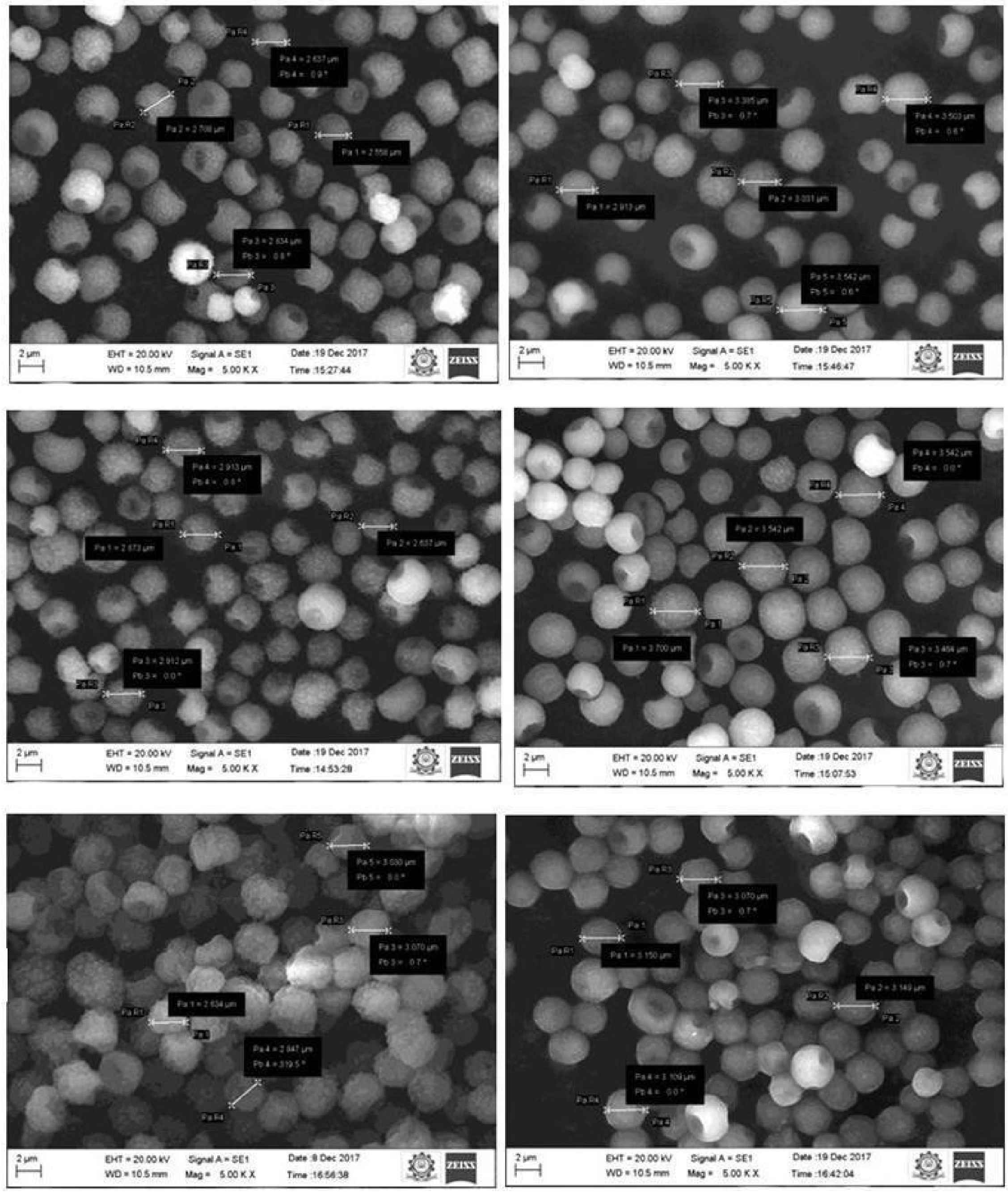

Supplementary Fig. S1. **SEM analysis of the three *A. flavus* strains ATCC 200026, MTCC 13369, and MTCC 13368 conidia (A) Melanised conidia, (B) Non-melanised conidia.** The diameter of conidia was measured using Image J software, and the mean diameter and SD were calculated (Data given in Table S1).

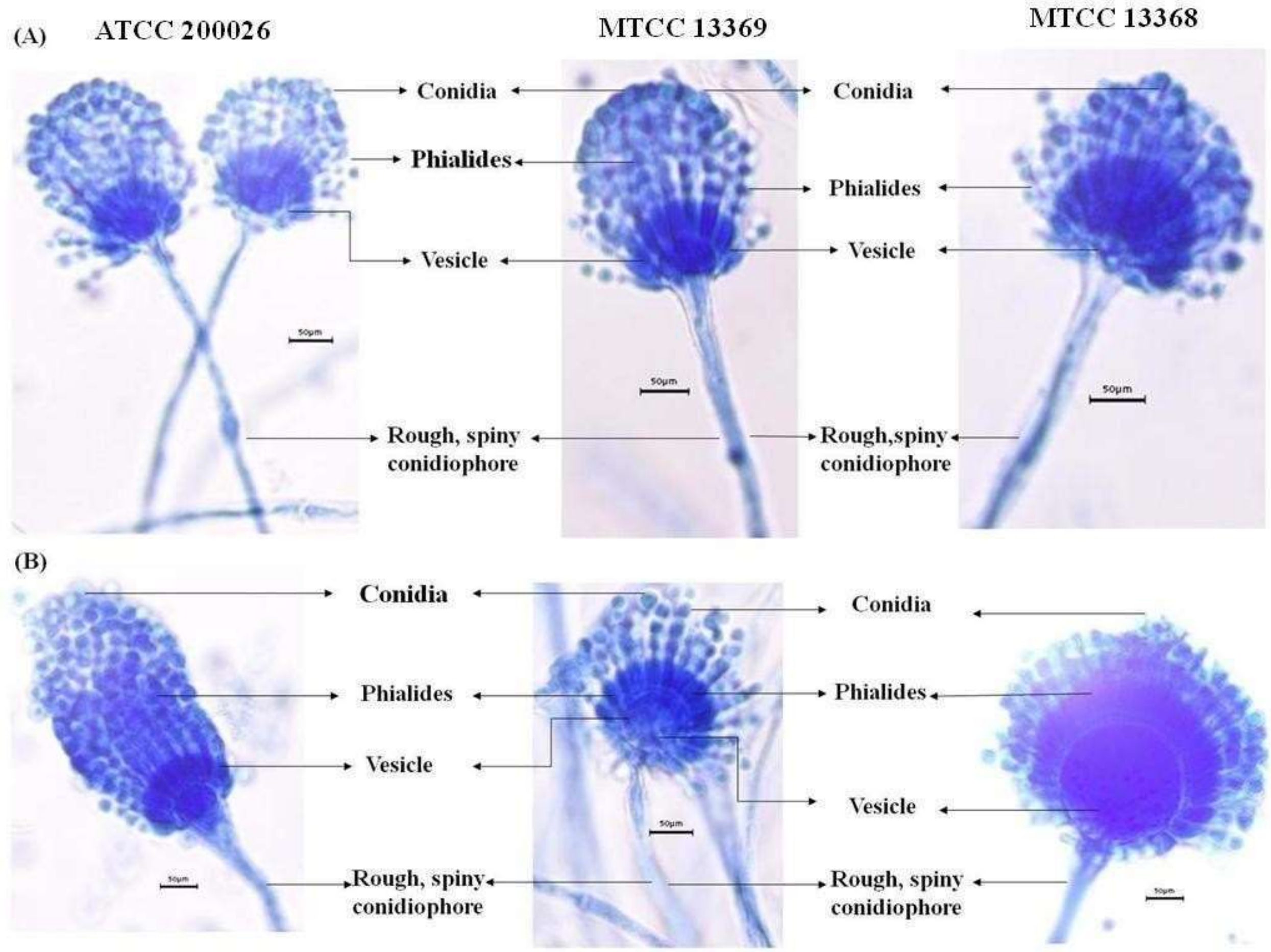

Supplementary Fig. S2. **Microscopic image of lactophenol cotton blue stained *A. flavus* conidia.** Various structures of the treated and untreated *A. flavus* strains ATCC 200026, MTCC 13369, and MTCC 13368 were unaltered showing that the kojic acid treatment and the absence of melanin did not affect the growth (A) Melanised conidia and of (B) Non-melanised conidia. Size bars are shown at the bottom of the figures (length of size bar: 50 μm).

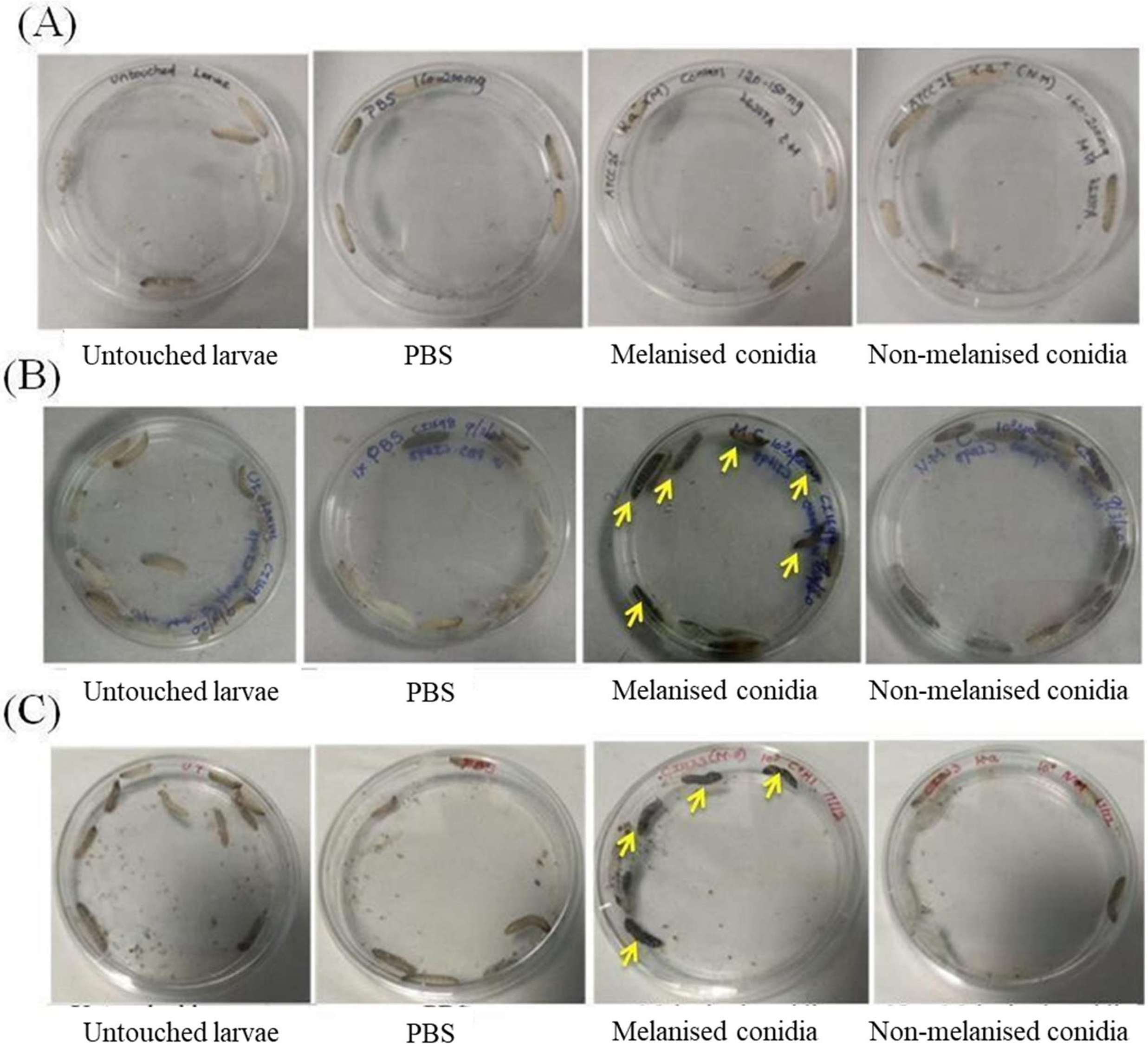

Supplementary Fig.S3. **In vivo virulence assay of saprophyte and clinical strains using *G. mellonella* larvae.** The 7th segment from the thorax is proleg (the injection point for this experiment), 10 larvae were administrated with 10 µl of 10^3 CFU (colony forming units)/ml of *A. flavus* conidia. PBS (phosphate-buffered saline) injected larvae, and the untouched larvae were used as the control groups. Larvae were kept at 37 °C for 3 days in sterile Petri plates. After 72 h post infection, black-colored larvae with no cocoon formation and movement were considered dead (yellow arrow), while cream-colored larvae with less cocoon formation and movement were considered alive. (A) ATCC 200026, (B) MTCC 13369, (C) MTCC 13368.

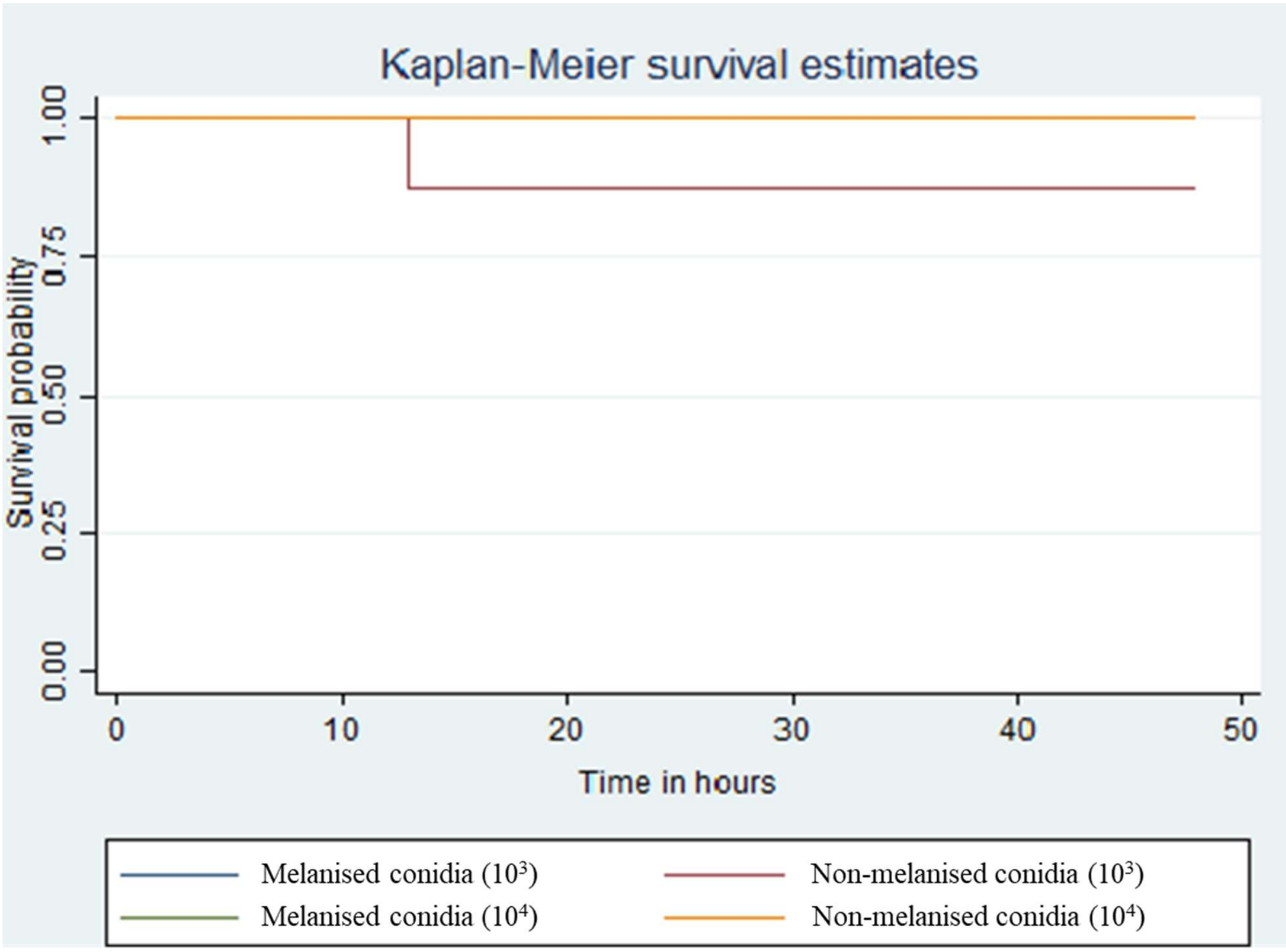

Supplementary Fig. S4. **Kaplan–Meier survival graph of the strain ATCC 200026**. The survival rate of larvae injected with MC and NMC was not significantly different. Only One dead larva (injected with 10^3/10 µl NMC) was detected during the 13-hour infection period.
